# Prevalence of concomitant rheumatologic diseases and autoantibody specificities among racial and ethnic groups in SLE patients

**DOI:** 10.3389/fepid.2024.1334859

**Published:** 2024-03-06

**Authors:** Brendan Denvir, Philip M. Carlucci, Kelly Corbitt, Jill P. Buyon, H. Michael Belmont, Heather T. Gold, Jane E. Salmon, Anca Askanase, Joan M. Bathon, Laura Geraldino-Pardilla, Yousaf Ali, Ellen M. Ginzler, Chaim Putterman, Caroline Gordon, Kamil E. Barbour, Charles G. Helmick, Hilary Parton, Peter M. Izmirly

**Affiliations:** ^1^ Department of Medicine, New York University Grossman School of Medicine, New York, NY, United States; ^2^ Division of Rheumatology, Department of Medicine, New York University Grossman School of Medicine, New York, NY, United States; ^3^ Department of Population Health, New York University Grossman School of Medicine, New York, NY, United States; ^4^ Division of Rheumatology, Department of Medicine, Hospital for Special Surgery, Weill Cornell Medical College, New York, NY, United States; ^5^ Division of Rheumatology, Department of Medicine, Columbia University College of Physicians & Surgeons, New York, NY, United States; ^6^ Division of Rheumatology, Department of Medicine, Icahn School of Medicine at Mount Sinai, New York, NY, United States; ^7^ Division of Rheumatology, Department of Medicine, SUNY Downstate Health Sciences University, Brooklyn, NY, United States; ^8^ Azrieli Faculty of Medicine, Zefat, Israel; ^9^ Rheumatology Research Group, Institute of Inflammation and Ageing, College of Medical and Dental Sciences, University of Birmingham, Birmingham, United Kingdom; ^10^ Division of Population Health, National Center for Chronic Disease Prevention and Health Promotion, Centers for Disease Control and Prevention, Atlanta, GA, United States; ^11^ Division of Disease Control, Bureau of Communicable Disease, New York City Department of Health and Mental Hygiene, Long Island City, NY, United States

**Keywords:** systemic lupus erythematosus, Sjögren's disease, antiphospholipid syndrome, fibromyalgia, autoantibodies

## Abstract

**Objective:**

Leveraging the Manhattan Lupus Surveillance Program (MLSP), a population-based registry of cases of systemic lupus erythematosus (SLE) and related diseases, we investigated the proportion of SLE with concomitant rheumatic diseases, including Sjögren’s disease (SjD), antiphospholipid syndrome (APLS), and fibromyalgia (FM), as well as the prevalence of autoantibodies in SLE by sex and race/ethnicity.

**Methods:**

Prevalent SLE cases fulfilled one of three sets of classification criteria. Additional rheumatic diseases were defined using modified criteria based on data available in the MLSP: SjD (anti-SSA/Ro positive and evidence of keratoconjunctivitis sicca and/or xerostomia), APLS (antiphospholipid antibody positive and evidence of a blood clot), and FM (diagnosis in the chart).

**Results:**

1,342 patients fulfilled SLE classification criteria. Of these, SjD was identified in 147 (11.0%, 95% CI 9.2–12.7%) patients with women and non-Latino Asian patients being the most highly represented. APLS was diagnosed in 119 (8.9%, 95% CI 7.3–10.5%) patients with the highest frequency in Latino patients. FM was present in 120 (8.9%, 95% CI 7.3–10.5) patients with non-Latino White and Latino patients having the highest frequency. Anti-dsDNA antibodies were most prevalent in non-Latino Asian, Black, and Latino patients while anti-Sm antibodies showed the highest proportion in non-Latino Black and Asian patients. Anti-SSA/Ro and anti-SSB/La antibodies were most prevalent in non-Latino Asian patients and least prevalent in non-Latino White patients. Men were more likely to be anti-Sm positive.

**Conclusion:**

Data from the MLSP revealed differences among patients classified as SLE in the prevalence of concomitant rheumatic diseases and autoantibody profiles by sex and race/ethnicity underscoring comorbidities associated with SLE.

## Introduction

Systemic lupus erythematosus (SLE) is a potentially fatal systemic autoimmune disease with heterogeneous clinical manifestations (1). SLE can occur as an isolated disease or coexist with other autoimmune diseases (2–4). The epidemiology of concomitant rheumatic diseases in SLE has been reported in several studies, with prevalence estimates varying greatly by study. For example, it is estimated that between 6% and 30% of SLE patients are also diagnosed with Sjögren's disease (SjD) (5–12), up to 30% of SLE patients are diagnosed antiphospholipid syndrome (APLS) (13–16) and between 12% and 27% are diagnosed with fibromyalgia (FM) (17–25). While several publications have reported these estimates, the literature is limited by smaller sample sizes that are not population-based and often do not address prevalence by race and ethnicity.

A hallmark of SLE is the presence of autoantibodies against a myriad of self-antigens, many being complexed with nucleic acids. Autoantibody specificities in SLE can be highly dynamic, such as anti-dsDNA, or relatively constant, such as those reactive with ribonuclear proteins, also referred to as extractable nuclear antigens (ENAs). Autoantibodies often provide insights into disease pathogenesis and may assist with diagnosis and prognosis (26, 27). Several studies investigating ENA positivity in SLE have identified racial and ethnic differences (16, 28). For example, across several studies, anti-Smith seropositivity is more common in Black patients compared with White patients (16, 29–35). In addition to ENAs, patients with SLE can have autoantibodies to phospholipids which increase thrombotic risk (1, 13–16). Several studies suggest the prevalence of anti-phospholipid antibodies among SLE patients to be between 20% and 33% (36, 37); however, much of these data are often limited by single race cohorts, and differences between races in concomitant APLS are not well understood.

SLE has a higher incidence among people from racial and ethnic minority populations, and these patients tend to suffer from worse outcomes (1, 28, 38, 39). These trends persist even after adjusting for socioeconomic status (28). Accordingly, knowledge of clinical phenotypes extending beyond primary SLE and distribution of antibody specificities among people from racial and ethnic minority groups should further contribute to the understanding of health disparities. While existing literature provides some insights into this area, most data are derived from cohort-based studies. The Manhattan Lupus Surveillance Program (MLSP) is a Centers for Disease Control and Prevention (CDC) funded population-based registry that recorded detailed clinical data on the large, multiracial/ethnic population of patients with SLE and related diseases living in Manhattan (1, 40–42). This study leveraged the MLSP to further investigate the proportion of concomitant SjD, APLS, and FM among SLE patients and to report the prevalence of specific autoantibodies in SLE by sex and race/ethnicity.

## Methods

A detailed overview of the MLSP has been previously reported (1). Briefly, data were extracted from medical records under the health surveillance exemption to HIPAA privacy rules [45 CFR § 164.512(b)] and as authorized by New York City Charter Sections 556(c)(2) and (d)(2). As a surveillance study, no cases were contacted for this study, and the creation of the MLSP did not require institutional review board (IRB) approval at CDC, New York City Department of Health and Mental Hygiene (DOHMH), or the New York University Grossman School of Medicine. The DOHMH IRB approved secondary analyses, including those reported herein, on a de-identified dataset (NYC DOHMH IRB no. 16–147). The surveillance period was from 1 January 2007 through 31 December 2009 in Manhattan. Manhattan was more diverse than the USA overall based on the 2010 census data with 48% of the population being non-Latino White, 25% Latino, 13% non-Latino Black and 11% non-Latino Asian residents (43).

### Case finding, data collection, and quality control of data entry

Case-finding sources included rheumatology practices (both adult and pediatric), hospitals, and hospitalization discharge and death registry databases (1). Sources were queried retrospectively to identify patients who lived in Manhattan with the following International Classification of Disease Ninth Revision Clinical Modification (ICD-9CM) billing codes: 710.0 (SLE) (1), 695.4 (discoid lupus erythematosus) (41), 710.8 (other specified connective tissue disease which is often used for mixed connective tissue disease) (42), 710.9 (unspecified connective tissue disease), and 710.2 (Sicca syndrome, which is used for SjD) (40). Every patient who lived in Manhattan with one of these ICD-9CM codes had their chart abstracted; the final diagnosis and date of diagnosis were coded by trained abstractors with medical degrees who underwent extensive training and routine quality assurance. Overall, 90.5% of hospitals and 75.8% of rheumatologists’ practices were included in the MLSP (1).

Data elements collected included common autoantibodies that were part of the three sets of classification criteria for lupus, including antinuclear antibody (ANA), anti-double stranded DNA (anti-dsDNA) antibody, anti-Smith antibody, and the anti-phospholipid (aPL) antibodies. In addition, common autoantibodies not part of classification criteria were also collected, including anti- Sjögren's Syndrome A (anti-SSA/Ro) and anti- Sjögren's Syndrome B (anti-SSB/La). These were considered positive if a patient ever had a positive result and negative if a patient had equivocal or borderline results. Since not all patients had evidence of testing, a result was considered negative only if there was information that the test had been performed. Likewise it was clearly noted if the specific laboratory tests were not found in the medical chart. A physician report of a positive test was accepted even if the primary laboratory tests were not found. Lupus anticoagulant was only included if the screen and confirmatory tests were positive. Anti-cardiolipin IgG and IgM were only considered positive if medium or high titer (>40 GPL/MPL units). Anti-beta 2 glycoprotein I IgG and IgM were considered positive by lab results, with equivocal or borderline results considered negative. We combined results and reported if any of the five individual antiphospholipid antibodies (lupus anticoagulant, anti-cardiolipin IgG and IgM antibodies, and anti-β2 glycoprotein I IgG and IgM antibodies) were positive at least once.

### Case definitions

SLE was defined as meeting at least one of the following: 1) 1997 American College of Rheumatology (ACR) (44), Systemic Lupus Erythematosus Collaborating Clinics (SLICC) (45), or 2019 European League Against Rheumatism (EULAR)/ACR classification criteria (46) as we previously described (47). Concomitant rheumatic diseases were defined using modified criteria based on available data collected in the MLSP: SjD (anti-SSA/Ro positive and evidence of keratoconjunctivitis sicca and/or xerostomia, which was our restrictive definition in the primary SjD analysis) (40), and APLS (antiphospholipid antibody positive and evidence of an arterial or venous clot). FM was defined by chart diagnosis. A separate analysis was conducted which relied only on a physician's stated diagnosis of SLE with SjD, APLS, and FM that was not restricted to any classification criteria.

### Statistical analysis

The proportion of SLE cases associated with each outcome and with autoantibody seropositivity were calculated by sex and race/ethnicity. Univariate differences were evaluated using chi-square tests or Fisher's exact tests when necessary. Bonferoni correction was applied for the ANA subtypes (anti-dsDNA abs, anti-Sm abs, Anti SSA/Ro abs, and anti-SSB/La abs) and only those with *p* values <0.0125 remained significant. For differences by race/ethnicity, comparisons excluded cases categorized as non-Latino other or unknown as being too small to evaluate. Analyses were also performed to understand whether there were differences in laboratory testing found in the chart. Sensitivity analyses were performed to determine the impact of missing antibody data on our findings on overlapping SjD and APLS.

## Results

### Concomitant diagnoses

Of the 1,342 SLE patients included in the study 1,226 (91.4%) were women; 400 (29.8%) Latino; 394 (29.4%) non-Latino White; 343 (25.6%) non-Latino Black; 148 (11.0%) non-Latino Asian; and 57 (4.2%) non-Latino Other. The average age of the SLE patients was 44 years. Of the 1342 cases, 1079 met the 1997 ACR classification criteria, 1265 met the SLICC classification critiera and 1029 met the EULAR/ACR criteria. Clinical criteria of the 1342 patients are included in Table 1.

**Table 1:** Clinical criteria among 1342 prevalent SLE cases meeting the 1997 ACR, SLICC, or EULAR/ACR classification criteria.

Clinical Criteria	*N*	%
Lymphopenia	1,002	74.7
Arthritis	923	68.8
Leukopenia	688	51.3
Alopecia	564	42.0
Low C3	560	41.7
Low C4	535	39.9
Nephritis	531	39.6
Low complement	522	38.9
Serositis	480	35.8
Malar rash	441	32.9
Photosensitivity	387	28.8
Mucosal ulcers	350	26.1
Thrombocytopenia	281	20.9
Cranial and/or peripheral neuropathy	204	15.2
Discoid rash	194	14.5
Seizures	171	12.7
Psychosis	97	7.2
Maculopapular rash	79	5.9
Hemolytic anemia	57	4.2
Subacute cutaneous LE	36	2.7
Panniculitis	31	2.3
False-positive syphilis test	24	1.8
Acute confused state	18	1.3
Mononeuritis multiplex	15	1.1
Bullous skin lesions	12	0.9
Transverse myelitis	7	0.5
Chilblain lupus	4	0.3
Lupus tumidus	4	0.3

For SjD, 147 [11.0%, 95% confidence interval (CI) 9.2%–12.7%] fulfilled our criteria (Table 2), with a higher proportion among women (11.7% vs. 3.4%, *p *= 0.0046). In addition, there were racial/ethnic differences in SjD, with non-Latino Asian patients having the highest proportion (21.0%, 95% CI 14.2–29.7).

**Table 2:** Proportion of Sjögren's disease among Manhattan residents with SLE by sex and race/ethnicity.

	Total SLE, *n*	Sjögren's disease, *n* (%, [95% confidence interval)	*p*-value^a^
Overall	1,342	147 [11.0% (9.2–12.7%)]	
Sex			0.0046
Male	116	4 [3.4% (0.9%–8.8%)]	
Female	1,226	143 [11.7% (9.8%–13.6%)]	
Race/ethnicity^b^			0.0002
Non-Latino White	394	46 [11.7% (8.6%–15.6%)]	
Non-Latino Black	343	25 [7.3% (4.7%–10.8%)]	
Latino	400	43 [10.8% (7.8%–14.5%)]	
Non-Latino Asian	148	31 [21.0% (14.2%–29.7%)]	
Non-Latino other/unknown	57		

^a^ Difference in proportion of Sjögren's disease by sex using Fisher's exact test and by race/ethnicity were evaluated using chi-square tests.

^b^ Patients with unknown race were excluded from analyses by race.

APLS was seen in 119 (8.9%, 95% CI 7.3%–10.5%) patients with SLE (Table 3), and although no differences were seen between men and women, there were differences by race/ethnicity, with Latino patients having the highest proportion (12.3% 95% CI 9.1%–16.2%).

**Table 3:** Proportion of antiphospholipid syndrome among Manhattan residents with SLE by sex and race/ethnicity.

	Total SLE, *n*	Antiphospholipid syndrome, *n* [% (95% confidence interval)]	*p*-value^a^
Overall	1,342	119 [8.9% (7.3%–10.5%)]	
Sex			0.2044
Male	116	14 [12.1% (6.6%–20.3%)]	
Female	1,226	105 [8.6% (6.9%–10.2%)]	
Race/ethnicity^b^			0.0317
Non-Latino White	394	36 [9.1% (6.4%–12.7%)]	
Non-Latino Black	343	24 [7.0% (4.5%–10.4%)]	
Latino	400	49 [12.3% (9.1%–16.2%)]	
Non-Latino Asian	148	8 [5.4% (2.3%–10.7%)]	
Non-Latino other/unknown	57		

^a^ Difference in proportion of antiphospholipid syndrome by sex were evaluated using chi-square tests and by race/ethnicity using Fisher's exact test.

^b^ Patients with unknown race were excluded from analyses by race.

Finally, FM was diagnosed in 120 (8.9%, 95% CI 7.3%–10.5%) patients who met SLE criteria (Table 4), with women having a higher proportion than men (9.6% vs. 1.7%, *p *= 0.0018). There were also racial/ethnic differences in patients diagnosed with FM, with non-Latino White patients being the most frequently diagnosed (13.5%, 95% CI 10.1%–17.6%).

**Table 4:** Proportion of fibromyalgia of among Manhattan residents with SLE by sex and race/ethnicity.

	Total SLE, *n*	Fibromyalgia, *n* [% (95% confidence interval)]	*p*-value^a^
Overall	1,342	120 [8.9% (7.3%–10.5%)]	
Sex			0.0018
Male	116	2 [1.7% (0.2%–6.2%)]	
Female	1,226	118 [9.6% (7.9%–11.4%)]	
Race/ethnicity^b^			<0.0001
Non-Latino White	394	53 [13.5% (10.1%–17.6%)]	
Non-Latino Black	343	17 [5.0% (2.9%–7.9%)]	
Latino	400	40 [10.0% (7.1%–13.6%)]	
Non-Latino Asian	148	7 [4.7% (1.9%–9.8%)]	
Non-Latino other/unknown	57		

^a^ Difference in proportion of fibromyalgia by sex and by race/ethnicity were evaluated using Fisher's exact tests.

^b^ Patients with unknown race were excluded from analyses by race.

For each disease, similar patterns were seen when using a physician's clinical diagnosis and not restricting to criteria (data not shown). Sensitivity analyses for SjD and APLS which excluded missing antibody resulted in similar findings, though proportions with each outcome increased given the smaller denominator with complete information available, Supplementary Tables S1A,B.

### Proportion of autoantibodies in SLE patients

Overall, 1,285 (95.8%) patients who fulfilled at least one of the three sets of classification criteria for the assignment of SLE had results of an ANA test reported in the chart, 1,222 (95.1%, 95% CI 89.8%–100.4%) of which were positive. ANA positivity did not differ by sex or race/ethnicity. For anti-dsDNA antibodies, results were found for 1,231 (91.7%) patients, 678 (55.1%, 95% CI 50.9%–59.2%) of which were positive. In contrast to the presence of an ANA, anti-dsDNA antibodies significantly differed by race (*p *= 0.0005) and were most frequent in non-Latino Asian patients (62.3%, 95% CI 49.9%–77.0%) and least frequent in non-Latino White patients (46.5%, 95% CI 39.5%–53.4%), Table 5.

**Table 5:** Proportion of select autoantibodies among Manhattan residents with SLE by sex and race/ethnicity.

		Overall	Sex			Race/ethnicity^a^					
			Female	Male	*p*-value*	Non-Latino White	Non-Latino Black	Latino	Non-Latino Asian	Non-Latino other/unknown	*p*-value*
ANA	*N*	1,285	1,170	115		383	323	386	140	53	
	Pos [95% CI]	1,222 [95.1% (89.8%–100.4%)]	1,116 [95.4% (89.8%–101.0%)]	106 [92.2% (74.6%–109.7%)]	0.1684	360 [94.0% (84.3%–103.7%)]	308 [95.5% (84.7%–106.0%)]	369 [95.6% (85.8%–105.4%)]	135 [96.4% (80.2%–112.7%)]		0.6561
Anti-dsDNA abs^b^	*N*	1,231	1,128	103		366	304	374	138		
	Pos [95% CI]	678 [55.1% (50.9%–59.2%)]	610 [54.1% (49.8%–58.4%)]	68 [66.0% (51.3%–83.7%)]	0.0197	170 [46.5% (39.5%–53.4%)]	176 [57.9% (49.3%–66.4%)]	222 [59.4% (51.5%–67.2%)]	86 [62.3% (49.9%–77.0%)]	49	0.0005
Anti-Sm abs^b^	*N*	970	892	78		303	235	287	103		
	Pos [95% CI]	253 [26.1% (22.9%–29.3%)]	223 [25.0% (21.7%–28.3%)]	30 [38.5% (26.0%–54.9%)]	0.0094	37 [12.2% (8.6%–16.8%)]	88 [37.5% (30.0%–46.1%)]	82 [28.6% (22.7%–35.5%)]	39 [37.9% (26.9%–51.8%)]	42	<0.0001
Anti-SSA/Ro abs^b^	*N*	1,039	955	115		339	241	302	115		
	Pos [95% CI]	430 [41.4% (37.5%–45.3%)]	395 [41.4% (37.3%–45.4%)]	35 [41.7% (29.0%–58.0%)]	0.9565	95 [28.0% (22.7%–34.3%)]	106 [44.0% (35.6%–52.4%)]	147 [48.7% (40.8%–56.5%)]	73 [63.5% (49.8%–79.8%)]	42	<0.0001
Anti-SSB/La abs^b^	*N*	1,018	938	80		335	233	297	111		
	Pos [95% CI]	205 [20.1% (17.4%–22.9%)]	192 [20.5% (17.6%–23.4%)]	13 (16.3% [8.7%–27.8%)	0.3664	43 [12.8% (9.3%–17.3%)]	40 [17.2% (12.3%–23.4%)]	78 [26.3% (20.8%–32.8%)]	36 [32.4% (22.7%–44.9%)]	42	<0.0001
aPL abs^c^	*N*	959	880	79		293	220	295	114		
	Pos [95% CI]	159 [16.6% (14.0%–19.2%)]	143 [16.3% (13.6%–18.9%)]	16 [20.3% (11.6%–32.9%)]	0.3594	66 [22.5% (17.4%–28.7%)]	23 [10.5% (6.6%–15.7%)]	50 [17.0% (12.6%–22.4%)]	15 [13.2% (7.4%–21.7%)]	57	0.0025

Only patients with antibody testing found in the chart were included in these analyses.

Abs, antibodies; aPL, antiphospholipid; ANA, antinuclear antibodies; CI, confidence interval.

^a^ Patients with unknown race were excluded from analyses by race.

^b^ After Bonferoni correction for the ANA subtypes only those *p* values <0.0125 remained significant.

^c^ Counted if any aPL were positive among those with valid data for at least one test.

* Differences in the proportion of autoantibodies by sex and by race/ethnicity were evaluated using chi-square tests.

Results for anti-Sm antibodies were found in 970 (72.2%) patients with 26.1% (95% CI 22.9%–29.3%) being positive. Men were more likely to be positive than women (38.5% vs. 25.0%, *p* = 0.0094), and racial/ethnic differences were similar to that of anti-dsDNA antibodies (*p *< 0.0001), with non-Latino Asian patients having the highest proportion (37.9%, 95% CI 26.9%–51.8%) and non-Latino White patients having the lowest proportion (12.2%, 95% CI 8.6%–16.8%), Table 5.

Anti-SSA/Ro and anti-SSB/La antibodies were positive in 41.4%, (95% CI 37.5%–45.3%) and 20.1%, (95% CI 17.4%–22.9%) of 1,039 (77.4%) and 1,018 (75.6%) SLE patients with data available, respectively. There were no differences in the proportion positive by sex for having both antibodies, but there were racial/ethnic differences for both (*p *< 0.0001), with non-Latino Asian patients having the highest rate, and non-Latino White patients having the lowest for anti-SSA/Ro and anti-SSB/La antibodies (Table 5). For combined aPL antibodies, there were no differences by sex, but racial/ethnic differences were present (*p *= 0.0025), with non-Latino White patients having the highest proportion (22.5.8%, 95% CI 17.4%–28.7%) and non-Latino Black patients having the lowest proportion (10.5%, 95% CI 6.6%–15.7%).

Finding evidence of laboratory testing differed by race/ethnicity for anti-SSA/Ro and anti-SSB/La antibodies. For anti-SSA/Ro and SSB/La antibodies, the proportion with testing found in the chart was highest among non-Latino White patients and lowest among non-Latino Black patients (Supplementary Table S1). Regarding combined aPL antibodies, the proportion with documentation for any individual test was lowest among non-Latino Black patients. However, in cases where clotting was present no differences were found (data not shown).

## Discussion

Analysis of the MLSP, a large multi-ethnic CDC-funded population-based registry, provided insight into differences by sex and race/ethnicity in concomitant rheumatic diseases and autoantibody profiles among patients with SLE. In 1,342 patients fulfilling SLE classification criteria, 11.0%, (95% CI 9.2–12.7) met our criteria for SjD, 8.9%, (95% CI 7.3%–10.5%) met our criteria for APLS, and 8.9%, (95% CI 7.3–10.5) carried a diagnosis of FM. The distribution of cases meeting criteria differed by race/ethnicity for SjD, APLS, and FM. Cases meeting criteria for SjD and FM also differed by sex, both being more common in women. These differences were also seen when using a clinical diagnosis irrespective of criteria. The distribution of autoantibody seropositivity differed by race/ethnicity for all autoantibodies evaluated, except ANA. Distribution of autoantibodies only differed by sex for anti-Sm.

The MLSP represents a large and diverse group of patients with SLE, with the prevalence of concomitant rheumatic diseases being largely consistent with prior studies (6–16, 20–25). In this analysis of the MLSP, we found the highest proportion of concomitant SjD with SLE among non-Latino White and non-Latino Asian patients which parallels findings from the MLSP analyzing primary SjD (40). In 2010, Baer et al. found that SLE with overlapping SjD was more prevalent in White women than in other demographics; however, few Latino and Asian patients were in their cohort (10). Other meta-analyses characterizing SLE with SjD offer limited racial and ethnic data (11, 12). While it is not clear why concomitant SjD is more prevalent in these groups, these findings serve to improve recognition of this condition among SLE patients in the future.

Regarding APLS, our study shows a higher proportion diagnosed among non-Latino White and Latino SLE patients compared with non-Latino Black and non-Latino Asian patients. Although not a direct comparison to our findings, a previous cohort study found ethnic differences in the incidence of arterial and venous thromboembolism in patients with SLE. Specifically, aPL antibodies and non-Chinese ethnicity were associated with venous thromboembolism (VTE) while Chinese ethnicity was associated with arterial clotting (16). In another study among SLE patients with VTE events, African American patients were less likely to have a clinically significant aPL profile compared with White patients (48). Given the potentially devastating consequences of thrombotic events, understanding which patients face higher risk is important for surveillance.

FM was present in SLE patients in our cohort at a lower rate than has been reported in other studies (17–25, 49). Interestingly, in our cohort, SLE with FM was more prevalent in non-Latino White and Latina women compared with other groups. Friedman et al. similarly found that SLE with concomitant FM was more prevalent in White women compared with Black women; however, their study included few Latina women (23). Because FM is a clinical diagnosis that does not necessarily require pharmacologic treatment, patients may not be universally screened during SLE visits. Without definite screening or required intervention, it is not surprising for chart-documented FM prevalence to be lower than expected. Since FM is a major contributor to decreased health-related quality of life among SLE patients suffering from this comorbidity, our findings highlight a need to increase screening to alleviate disease burden and identify potential target populations most at risk of co-morbid FM (50).

Analysis of autoantibody profiles in the MLSP offers interesting insights compared with similar analyses in prior studies. Anti-dsDNA was most prevalent in non-Latino Asian patients and high in non-Latino Black and Latino patients compared with non-Latino White patients—consistent with findings from other multi-ethnic cohorts (29, 34). Regarding anti-Sm antibodies, our study showed the highest proportion in non-Latino Black and Asian patients compared with Latino and non-Latino White patients. Several studies have demonstrated similar findings (29–34, 48). Additionally, significantly more Latino patients were anti-Sm positive compared with non-Latino White patients, which is also consistent with prior studies (29, 31, 51). Regarding anti-SSA/Ro and anti-SSB/La antibodies, both were most prevalent in non-Latino Asian patients and least prevalent in non-Latino White patients in our cohort. Similar observations were found in a cohort study of newly diagnosed SLE patients with anti-SSA/Ro antibodies which were present in 57% of Chinese patients compared with 28% of African American patients and 18% of Caucasians (48). Recent studies have linked genetic ancestry to the heterogeneous gene expression and immunologic profiles in SLE. Our population level data may help inform future research in this area (52, 53). In addition, by better understanding how autoantibody profiles differ in racial/ethnic groups, we can more appropriately incorporate our patients’ serologic data in their clinical context.

In addition to differences between racial/ethnic groups, our study demonstrated several differences between sexes in both concomitant rheumatic diseases and autoantibody profiles. Men with SLE in our cohort were less likely to have SjD and FM compared with women. These differences have been demonstrated in previous literature for both secondary and primary SjD and FM (10, 20, 40). Regarding autoantibodies, men in our study were more likely to be anti-Sm positive. These differences have also been demonstrated previously (54).

There are several limitations inherent to using the MLSP registry described previously (1). The data used were from 2007 to 2009 but were unique in offering a large registry to study demographic associations in further detail. The data in the MLSP relies on chart documentation, and due to the heterogeneity of the medical records from which the data were abstracted, there is potential for missing data not present in the records accessed, such as antibody testing. In addition, medical care can vary and it is possible data such as keratoconjunctivitis sicca and/or xerostomia symptoms were not solicited by the treating physicians. In 4.2% of patients race/ethnicity was unknown. Differences were detected in the frequencies of laboratory testing with non-Latino Black patients having several antibodies, including anti-SSA/Ro and SSB/La antibodies and individual aPL tests, less likely to be found in the chart. Given the surveillance nature of the MLSP the etiology of these differences cannot be determined. The clinical relevance of the differences in frequency of some of the autoantibodies as a function of race/ethnicity is unknown. The definition of concomitant SjD required anti-SSA/Ro and the presence of keratoconjunctivitis sicca and/or xerostomia leaving the possibility that the differences found in the MLSP between race/ethnicity reflected a greater likelihood that non-Latino White patients were tested and clinically evaluated for keratoconjunctivitis sicca and/or xerostomia symptoms. In addition, our definition of SjD would not capture antibody negative disease. Although there were also differences in the frequencies of testing for individual aPLs, the presence of any thrombotic event prompted testing regardless of race/ethnicity. Finally, we acknowledge that inherent biases could influence our results. For example, physicians may have biases regarding the evaluation of FM in males since the condition is more common in women.

Individual autoantibody data in patients meeting our SLE case definition were incomplete. While we considered equivocal antibodies negative, any positive titer was counted regardless of level of positivity. It is acknowledged that we cannot account for the variation in assays used for antibody testing. This is a limitation, as different assays have different sensitivities and specificities, and accounting for these differences may impact the results. It is also acknowledged that since antibodies, particularly anti-dsDNA and aPL, can fluctuate over time and with disease activity, a positive test at a given point in time may have been missed resulting in a potential underrepresentation of the frequency. In this study, we use modified classification criteria for SjD and APLS, which may not accurately capture these diseases. However, separate analyses looking at diagnoses that are not criteria-based and sensitivity analyses accounting for missing antibody data found similar findings.

The study has several strengths as it used a large population-based registry comprising a multi-racial/ethnic composition to allow analyses by race/ethnicity. The MLSP also included a significant number of male SLE patients to allow for differences to be detected by sex. This study offers interesting insights into the nature of SLE by showing associations of race/ethnicity with coexisting rheumatic disease and autoantibody profiles. The results of this study highlight the importance of delineating co-morbid conditions and measuring autoantibodies in all SLE patients. As SLE is a heterogeneous disease, characterizing a patient’s phenotype is important for understanding their risk profile and their trajectory.These findings open the door for future research directed at answering the questions of why differences exist between demographic groups, and how these differences relate to observed differences in clinical outcomes.

## Data Availability

The data analyzed in this study is subject to the following licenses/restrictions: This study used data from a surveillance initiative which are housed at the NYC DOHMH. Requests to access these datasets should be directed to hparton@health.nyc.gov.
