# Supplementary material for: Prevalence of concomitant rheumatologic diseases and autoantibody specificities among racial and ethnic groups in SLE patients
Source: Front Epidemiol. 2024 Mar 6;4:1334859. doi: 10.3389/fepid.2024.1334859 (PMC10956350; doi:10.3389/fepid.2024.1334859)
Supplement: Supplementary file 1 [file Table1.docx]

| **Supplemental Table 1A: Proportion of Sjögren’s disease among Manhattan residents with SLE by sex and race/ethnicity excluding cases with missing antibody data** | | | |
| --- | --- | --- | --- |
|  | **Total SLE, n** | **Sjögren’s Disease, n (%, [95% confidence interval)** | ***p*-value*** |
| **Overall** | 995 | 147 (14.8% [12.4-17.2%]) |  |
| **Sex** |  |  | **0.0025** |
| Male | 82 | 4 (4.9% [1.3-12.5%]) |  |
| Female | 913 | 143 (15.7% [13.1-18.2%]) |  |
| **Race/ethnicity**** |  |  | **0.0005** |
| Non-Latino White | 324 | 46 (14.2% [10.4-18.9%]) |  |
| Non-Latino Black | 232 | 25 (10.8% [7.0-15.9%]) |  |
| Latino | 287 | 43 (15.0% [10.8-20.2%]) |  |
| Non-Latino Asian | 111 | 31 (27.9% [19.0-39.6%]) |  |
| Non-Latino  other/unknown | 41 |  |  |

* Difference in proportion of Sjögren’s disease by sex using Fisher’s exact test and by race/ethnicity were evaluated using chi-square tests.

** Patients with unknown race were excluded from analyses by race.

| **Supplemental Table 1B: Proportion of antiphospholipid syndrome** **among Manhattan residents with SLE by sex and race/ethnicity excluding cases with missing antibody data** | | | |
| --- | --- | --- | --- |
|  | **Total SLE, n** | **Antiphospholipid Syndrome,**  **n (% [95% confidence interval])** | ***p*-value*** |
| **Overall** | 959 | 119 (11.8% [9.6-14.0%]) |  |
| **Sex** |  |  | 0.3269 |
| Male | 79 | 14 (15.2% [7.9-26.5%]) |  |
| Female | 880 | 105 (11.5% [9.2-13.7%]) |  |
| **Race/ethnicity**** |  |  | **<0.0001** |
| Non-Latino White | 293 | 36 (11.3% [7.8-15.8%]) |  |
| Non-Latino Black | 220 | 24 (10.9% [7.0-21.4%]) |  |
| Latino | 295 | 49 (15.9% [11.7-21.2%]) |  |
| Non-Latino Asian | 114 | 8 (7.0% [3.0-13.8%]) |  |
| Non-Latino other/unknown | 37 |  |  |

* Difference in proportion of antiphospholipid syndrome by sex were evaluated using chi-square tests and by race/ethnicity using Fisher’s exact test.

** Patients with unknown race were excluded from analyses by race.

**Supplemental Table 2: Evidence of testing among prevalent SLE cases residing in Manhattan 2007-2009, by race/ethnicity**

|  | Non-Latino White   patients  N (%) | Non-Latino Black   patients  N (%) | Latino  patients  N (%) | Non-Latino Asian  patients  N (%) | p-value |
| --- | --- | --- | --- | --- | --- |
| **ANA** |  |  |  |  | 0.1439 |
| Evidence of testing found | 383 (97.2) | 323 (94.2) | 386 (96.5) | 140 (94.6) |  |
| Evidence of testing not found | 11 (2.8) | 20 (5.8) | 14 (3.5) | 5.4 (5.4) |  |
| **Anti-DNA** |  |  |  |  | 0.0646 |
| Evidence of testing found | 366 (92.9) | 304 (88.6) | 374 (93.5) | 138 (93.2) |  |
| Evidence of testing not found | 28 (7.1) | 39 (11.4) | 26 (6.5) | 10 (6.8) |  |
| **Anti-Sm** |  |  |  |  | 0.0655 |
| Evidence of testing found | 303 (76.9) | 235 (68.5) | 287 (71.8) | 103 (69.6) |  |
| Evidence of testing not found | 91 (23.1) | 108 (31.5) | 113 (28.3) | 45 (30.4) |  |
| **Anti-SSA/Ro** |  |  |  |  | <0.0001 |
| Evidence of testing found | 339 (86.0) | 241 (70.3) | 302 (75.5) | 115 (77.7) |  |
| Evidence of testing not found | 55 (14.0) | 102 (29.7) | 98 (24.5) | 33 (22.3) |  |
| **Anti-SSB/La** |  |  |  |  | <0.0001 |
| Evidence of testing found | 339 (86.0) | 233 (67.9) | 297 (74.3) | 111 (75.0) |  |
| Evidence of testing not found | 55 (14.0) | 110 (32.1) | 103 (25.8) | 37 (25.0) |  |
